# Defining the Reliability of Deltoid Reanimation by Nerve Transfer When Using Abnormal but Variably Recovered Triceps Donor Nerves

**DOI:** 10.3389/fsurg.2021.691545

**Published:** 2021-06-28

**Authors:** Scott Ferris, Aaron Withers, Lipi Shukla

**Affiliations:** ^1^Plastic, Hand and Faciomaxillary Surgery, The Alfred Hospital, Prahan, VIC, Australia; ^2^Department of Plastic and Reconstructive Surgery, St. Vincent's Private Hospital, East Melbourne, VIC, Australia

**Keywords:** brachial, plexus, deltoid, reanimation, triceps, nerve transfer

## Abstract

Upper brachial plexus injuries to the C5/6 roots or axillary nerve can result in severe deficits in upper limb function. Current techniques to reinnervate the deltoid muscle utilise the well-described transfer of radial nerve branches to triceps to the axillary nerve. However, in around 25% of patients, there is a failure of sufficient deltoid reinnervation. It is unclear in the literature if deltoid reanimation should be attempted with a nerve transfer from a weak but functioning triceps nerve. The authors present the largest series of triceps to axillary nerve transfers for deltoid reanimation in order to answer this clinical question. Seventy-seven consecutive patients of a single surgeon were stratified and analysed in four groups: (1) normal triceps at presentation, (2) abnormal triceps at presentation recovering to clinically normal function preoperatively, (3) abnormal triceps at presentation remaining abnormal preoperatively, and lastly (4) where pre-operative triceps function was deemed insufficient for use, requiring alternative reconstruction for deltoid reanimation. The authors considered deltoid re-animation of ≥ M4 as successful for the purpose of this study. Median Medical Research Council (MRC) values demonstrate group 1 achieves this successfully (M5), while median values for groups 2–4 result in M4 power (albeit with decreasing interquartile ranges). Median post-operative shoulder abduction active range of motion (AROM) values were represented by 170° (85–180) in group 1, 117.5° (97.5–140) in group 2, 90° (35–150) in group 3, and 60° (40–155) in group 4. For both post-operative assessments, subgroup analyses demonstrated statistically significant differences when comparing group 1 with groups 3 and 4 (*p* < 0.05), while all the other group to group pairwise comparisons did not reach significance. The authors postulated that triceps deficiency can act as a surrogate marker of a more extensive plexus injury and may predict poorer outcomes if the weakness persists representing the trending differences between groups 2 and 3. However, given no statistical differences were demonstrated between groups 3 and 4, the authors conclude that utilising an abnormal triceps nerve that demonstrates sufficient strength and redundancy intraoperatively is preferable to alternative transfers for deltoid reanimation. Lastly, in group 4 patients where triceps nerves are damaged and unusable for nerve transfer, alternative operations can also achieve sufficient outcomes and should be considered for restoration of shoulder abduction.

## Introduction

Injury to the upper brachial plexus (C5/C6 roots) or the axillary nerve results in devastating impairments of the function and aesthetics of the upper limb (1). The deltoid muscle is innervated by the axillary nerve from the posterior cord of the brachial plexus and is traditionally thought to be the main shoulder abductor. It is assisted by supraspinatus, innervated by the suprascapular nerve (SSN) from the upper trunk of the brachial plexus, which initiates abduction and braces the head of the humerus to the glenoid fossa to provide stability during this motion (2). To restore shoulder function, the current options for treatment include nerve transfer, nerve grafting, tendon transfer, and arthrodesis of the glenohumeral joint (3).

Leechavengvongs et al. popularised the nerve transfer for re-animation of the injured deltoid muscle for shoulder abduction using the nerve to the long head of triceps to the anterior branch of the axillary nerve (4). This technique refined previous descriptions by Lurje (5) in the setting of a patient with Erb's palsy and a larger group reported by Nath and MacKinnon (6). Leechavengvongs described a posterior approach with nerve transfer preferentially of the long head of triceps due to its proximity to the recipient, size match, and constant branching point (7). Khair et al. conducted an anatomical study on cadaver specimens to ascertain the axonal counts of nerves involved in this transfer, concluding that that nerve to long head of triceps has 2,302 axons, nerves to medial head has 2,198 and nerve to lateral head has 1,462. With regards to the recipient nerves in this transfer, the axonal count of the main axillary trunk was 7,887, the anterior division was 4,052 and the posterior division was 1,242 axons with the remainder innervating teres minor (8). This transfer is commonly done in combination with spinal accessory nerve (SAN) transfer to the SSN for reanimation of supraspinatus and infraspinatus muscles to provide shoulder abduction and external rotation, the importance of which was later confirmed in biomechanical studies by Crouch et al. (9).

The transfer of radial nerve triceps branches to the axillary nerve is now well-described as a successful technique and the current preferred option of many brachial plexus surgeons for reinnervation of the deltoid. Bertelli et al. reported on 10 patients with C5/C6 root avulsions who underwent nerve to the long or lateral head of triceps to axillary nerve, SAN to SSN, and ulnar nerve fascicle to biceps motor branch transfers. All patients achieved active shoulder abduction in 2 years with three having Medical Research Council (MRC) muscle power scale scores of M4 (4/5) and seven scoring M3 (10). Leechavengvongs et al. reported the outcomes of the same combination of nerve transfers in 15 patients, all of whom demonstrated the return of deltoid function with 13 patients scoring M4 and two scoring M3 for shoulder abduction along with a mean shoulder abduction of 115° (4, 7, 11). More recently, Wheelock et al. demonstrated the efficacy of nerve to triceps to axillary nerve transfers in isolated axillary nerve injuries in the setting of shoulder dislocation. Eight out of 10 patients achieved ≥ M3 shoulder abduction at a mean follow-up of 14.8 months (12).

Despite these reports of successful outcomes, Lee et al. observed that failure of meaningful deltoid reinnervation with this transfer reaches around 25% (13). Factors such as older age, comorbidities and greater delay from injury to surgery have been raised as potential factors contributing to failure; however, this is not consistently reported (13). Desai et al. presented a case series of 27 patients undergoing triceps to axillary nerve transfers for either isolated axillary nerve injury or other brachial plexus injury (BPI), finding improved shoulder abduction in 89% of patients with > M3 (3). However, on subgroup analysis, the level of injury, triceps nerve branch transferred, or timing of surgery did not demonstrate any significant differences in outcomes (3).

All nerve transfers require intact donor axons, with most stating a prerequisite donor strength of ≥ M4, yet this variable is not consistently reported (4–6, 10, 13). There is, however, a significant cohort of patients requiring deltoid reanimation who present with and continue to demonstrate abnormal triceps function in the pre-operative setting. This is common, for example, in patients with a C5/6/7 injury. It is not well-discussed in the literature whether the reanimation of the deltoid muscle should be attempted with a nerve transfer from an impaired but functioning triceps nerve or from an apparently fully recovered triceps nerve. Furthermore, the literature does not characterise the strength of triceps function, which is deemed adequate for a successful nerve transfer. The decision to use an abnormal nerve to triceps as a donor nerve for transfer is largely clinical and driven by intraoperative findings, without significant guidance from current literature.

This study, therefore, aims to investigate this pre-operative surgical dilemma in order to inform clinical decision making. The surgical outcomes of a single surgeon performing triceps to axillary nerve transfers are evaluated and compared within three clinically distinct groups of patients: (1) normal triceps at presentation, (2) abnormal triceps at presentation recovering to clinically normal function preoperatively, and (3) abnormal triceps at presentation remaining clinically abnormal preoperatively. The outcomes of the fourth group of patients with severely abnormal or absent triceps function was also analysed. The pre-operative triceps function of this group was deemed insufficient for use, therefore requiring alternative reconstruction for deltoid reanimation (see Table 1).

**Table 1:** Patient groups as determined by Medical Research Council grading of triceps strength presentation and pre-operative consultation.

**Group**	**MRC triceps presentation**	**MRC triceps pre-operative**	**Outcome**
1	5	5	Suitable for transfer
2	<5	5	Recovered and suitable for transfer
3	<5	≥4 but <5	Deemed sufficient to use for transfer
4	<5	≤3	Unsuitable–alternative surgery for deltoid reinnervation

## Methods

### Case Selection

The prospectively maintained database and clinical notes were examined for patients who had received nerve to triceps transfer for deltoid reanimation between 2007 and 2018 by a single surgeon working in private and public institutions in Melbourne, Australia. All the patients presented with brachial plexus palsies underwent surgical intervention for deltoid reanimation and had follow-up for an average of 33 months post-operatively.

### Data Collection

Pre-operative data collected included patient demographics, co-morbidities, injury demographics, level of brachial plexus injury, and pre-operative functional assessment of donor and recipient nerves. The patients were placed into groups according to their MRC score of triceps strength at both presentation and time of surgery (12) (Table 1). Ethics approval for data collection was obtained from the local governing body HREC LRR 061/16.

Two independent reviewers collated all operative notes to record the procedure performed, intraoperative assessment of nerve branches to triceps, and details of nerve coaptations. These groups were segregated by documented pre-operative triceps function as described (see group allocation). By measuring the functional outcomes of deltoid reanimation, the authors aim to answer the clinical question at hand: can abnormal triceps nerves be used for axillary nerve transfer successfully?

### Group Allocation

The patients were placed in four groups according to their presenting and pre-operative triceps assessment (see Table 1).

1) Normal triceps at presentation.
2) Abnormal triceps at presentation recovering to clinically normal triceps function pre-operatively.
3) Abnormal triceps at presentation remaining clinically abnormal at time of surgery.
4) Pre-operative triceps function deemed insufficient for use, therefore, alternative reconstruction deltoid reinnervation.

Specific to the patients allocated to group 3, the decision to proceed using an “abnormal triceps” was based on a pre-operative M4 (deemed a strong 4). This category is commonly referred to as a 4^+^/5 and is well-understood by clinicians as a real clinical subgroup and was described in an update in 1943 (14). This was assessed by the surgical team and hand therapists with the ultimate decision at the discretion of the senior surgeon (SF).

### Operative Procedure

**Figure 1:**
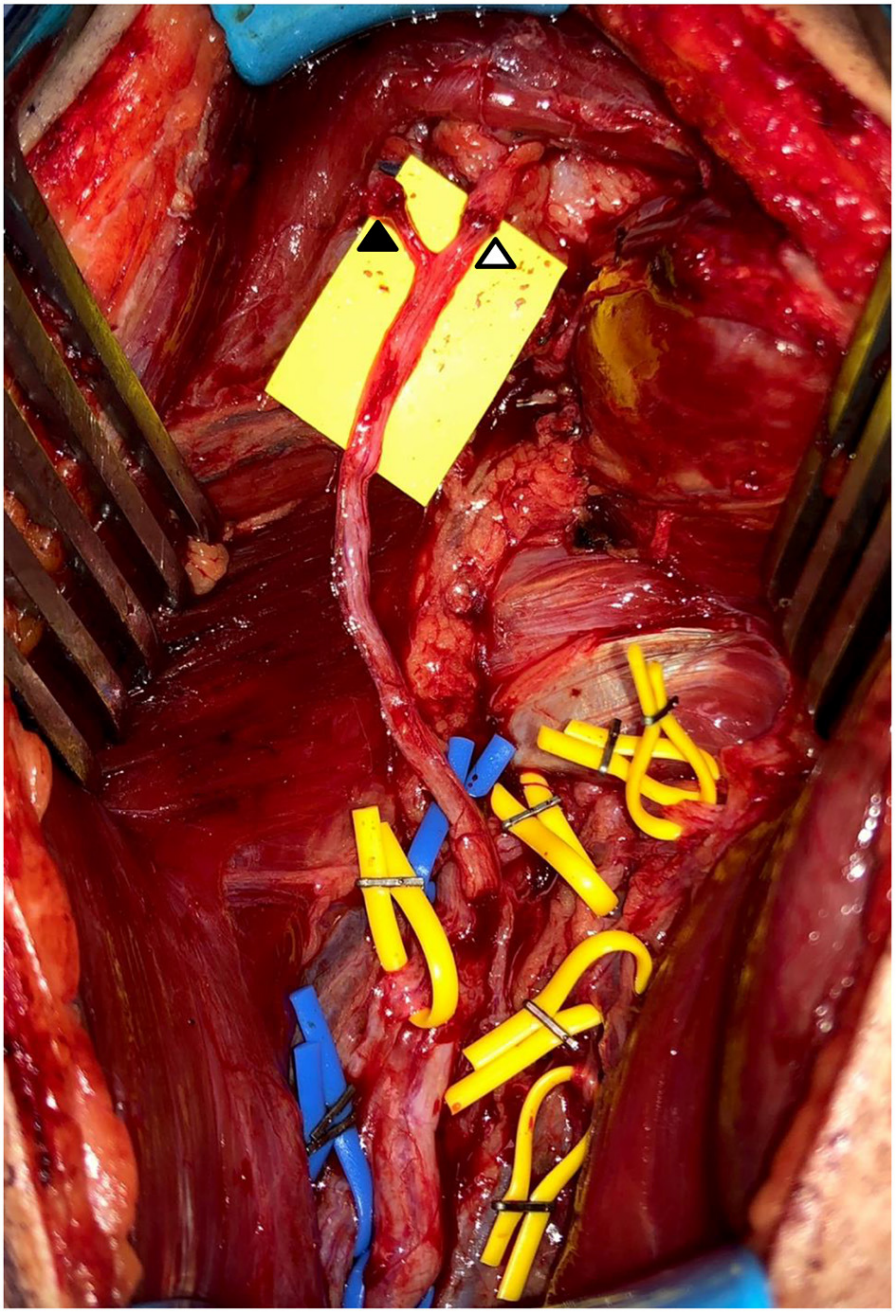
Intraoperative photography of triceps to axillary nerve transfers. Completed nerve transfers using medial head triceps nerves to both anterior motor axillary nerve (△) and motor component of posterior axillary nerve (▲). Dissected and preserved functioning nerves to triceps in yellow loops. Blue loops placed on non-stimulable sensory nerve and radial nerve proper.

The operative procedure performed was similar to that previously described by Leechavengvongs et al. (4). The patients were positioned in a lateral decubitus position and a posterior arm incision was made to gain access through the intermuscular cleft between the long and lateral heads of the triceps. Further dissection identifies the axillary nerve in the quadrangular space and the radial nerve and multiple triceps nerves in the triangular space (Figure 1). Neurolysis of the multiple (usually 7–10) nerves to the triceps was performed, followed by neurolysis of the branches of the axillary nerve.

Intraoperative stimulation offers different, and, in the practise of authors, much more useful information than pre-operative neurophysiological testing including EMG. The reason for this is because the essential questions in these patients become (a) are there enough sufficiently well-motored triceps nerves to use that will successfully reinnervate deltoid? and (b) if those nerves are sacrificed for the transfer, would there be sufficient residual triceps nerves for ongoing triceps function? This information is only possible to elicit by open dissection, then selective, insulated stimulation of each triceps nerve, which we routinely undertake at 0.5 mA. In this way, it is possible to accurately identify which triceps nerves are functioning and to what extent. This enables labelling those triceps nerves as either strongly functioning (excellent elbow extension when individually stimulated), weakly functioning (muscle seen to flicker but poor or no elbow extension) or non-functioning. Once this is complete, an assessment can be made as to whether there are sufficiently intact and strongly stimulating triceps nerves for both sacrifice to use in reconstruction while preserving good elbow extension. Generally, a minimum of three strongly stimulable triceps nerves (but usually more) are preserved for triceps function.

The procedure can safely be discontinued at this point if the evaluated donors demonstrate insufficient stimulation or insufficient redundancy to allow harvest while preserving adequately maintained triceps function. Once appropriate donors had been selected, donor neurotomies were undertaken as distally as required for tension-free transfers. The anterior axillary nerve was selectively the highest priority recipient nerve target. If motor components of intermediate or posterior axillary nerves were identified entering the deltoid muscle and sufficient donors were available, then further triceps to axillary nerve transfers were performed. Ultimately, it was most common for two or three (although less frequently one or four) triceps nerves to be transferred in any individual case. Coaptation was performed under the microscope without tension using 9-0 monofilament nylon and fibrin glue applied to the outside of completed nerve repairs. We have a preference to use medial head triceps nerves for reconstruction and preserve lateral and long head if possible but ultimately will use and leave whichever nerves are required in an individual case. The reason the senior author generally preferred using medial head nerves is because of their typically favourable length, calibre, and axon counts as well as their more concealed donor site contour deformity.

If a concurrent SAN to SSN transfer was planned, this was performed *via* a separate posterior shoulder approach during the same surgery. All the patients were placed in a shoulder immobiliser sling to protect nerve coaptations for 2 weeks. When reviewed at 2 weeks, the patients commenced mobilisation with the guidance of a hand therapist. Post-operative reviews were generally undertaken at ~3–6 monthly intervals thereafter.

### Post-operative Follow-Up

Post-operative outcomes included functional assessment comprising shoulder range of motion and MRC power grading. The time to plateau for a deltoid function was calculated as the time from surgery until the highest MRC shoulder abduction score or highest degree of shoulder abduction AROM was obtained and maintained at subsequent follow-up.

### Statistical Analyses

Continuous variables were summarised using mean (standard deviation) or median (interquartile range) according to data type and distribution. Categorical variables were reported as counts and proportions. Comparisons between groups were made by the Krukal–Wallis test for continuous variables and chi-square or Fisher's exact test as appropriate for categorical variables. *Post-hoc* pairwise comparisons were performed to determine differences among the four groups. A two-sided *p* < 0.05 was considered to be statistically significant. Analyses were performed with SAS software version 9.4 (SAS Institute, Cary, NC, United States).

## Results

Between the years 2007 and 2018, 80 patients presented to the senior author (SF) for surgical management of traumatic brachial plexus injuries requiring deltoid reanimation. Of the 80 patients, three were excluded because of insufficient pre- and post-operative assessment documentation or loss to follow-up. Therefore, the authors present outcomes for a series of 77 patients. Sixty-four patients underwent nerve to triceps transfer for deltoid reanimation, further subcategorised according to their triceps MRC grading: 45 patients (group 1) with normal triceps preoperatively, 5 patients (group 2) with abnormal triceps that improved to a clinically normal triceps preoperatively, and 14 (group 3) with abnormal triceps that improved although remained clinically abnormal preoperatively. The triceps nerve donors in group 3 patients, although not normal, were deemed to be sufficient for nerve transfer during pre-operative assessment and intra-operative interrogation. Using the on-table risk minimisation strategy outlined in Methods, no patient suffered any problematic or concerning post-operative downgrading of triceps power. Most commonly when available, multiple medial head triceps nerves would be used for the reconstruction. One reason for this is the favourable length of such nerves, such that they can be transferred very close to the entry to the muscle of motor axillary nerves, thereby reducing time to reinnervation. Another reason is that frequently these young, body-conscious patients prefer, if possible, to preserve the visible contour of the more proximal triceps musculature, particularly lateral head.

**Figure 2:**
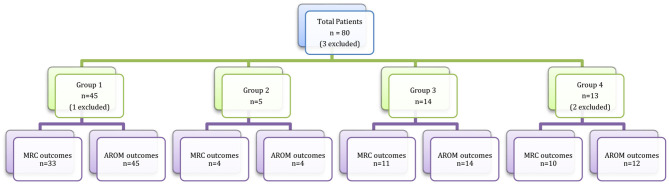
Total and subgroup patient numbers for outcome analysis.

The remaining 13 patients (group 4) underwent alternative nerve transfers for deltoid reanimation as they had no suitable triceps nerve donors. These operations included radial nerve fascicular transfers, thoracodorsal nerve transfers, intercostal nerve transfers to axillary nerve, or nerve grafting from a C5 root (Table 2). The breakdown of total patient number, subgroup numbers and numbers of patients with sufficiently recorded data for statistical outcome analysis is depicted in Figure 2.

**Table 2:** Alternative nerve transfers and grafting procedures performed for group 4 patients.

**Sex**	**Age**	**Operative procedure for deltoid reanimation**
F	24	Radial nerve interfascicular neurolysis. Transfer 2 fascicles (wrist extensors) to anterior axillary nerve.
M	55	Radial nerve interfascicular neurolysis. Transfer 2 fascicles (wrist extensors) to anterior axillary nerve.
M	51	Radial nerve interfascicular neurolysis. Transfer 1 fascicle (wrist extensors) to anterior axillary nerve.
M	29	Sural nerve cable graft C5 to axillary nerve
M	31	Sural nerve cable graft from distal SAN to axillary nerve.
M	26	Sural nerve cable graft posterior division upper trunk to axillary nerve
M	19	Sural nerve cable graft ICN 5/6/7 to axillary nerve
M	30	Sural nerve cable graft C5 to axillary nerve
M	22	Vascularised thoracodorsal nerve transfer to axillary nerve
M	37	Vascularised thoracodorsal nerve transfer to axillary nerve
M	32	Sural nerve cable graft C5 to axillary nerve
M	54	Sural nerve cable graft C5 to axillary nerve
M	23	Sural nerve cable graft from posterior cord to axillary nerve

The demographics of the 77 patients presented in this series demonstrate a mean age of 39.5 ± 4.4 years with a 90% male pre-dominance, with no significant differences between groups. The patterns of brachial plexus injury, as well as presentation and pre-operative triceps MRC grading, were significantly different and in keeping with the severity of the injury basis of group categorisation. The average time from injury to surgery was 6.5 ± 3.7 months across all groups with no significant difference between groups (Table 3).

**Table 3:** Patient and injury demographics.

	**Group 1**	**Group 2**	**Group 3**	**Group 4**
**PATIENT DEMOGRAPHICS**				
Total patients	45	5	14	13
Age at injury mean (std)	39.2 ± 16.3	42.1 ± 22.9	43.1 ± 17.3	33.3 ± 12.5
Gender Ratio M:F	39:6	5:0	13:1	12:1
**TRICEPS FUNCTION**				
Presentation Triceps MRC median (IR)	5 (5–5)	4 (3–4)	4 (4–4)	0 (0–2)
Pre-operative Triceps MRC median (IR)	5 (5–5)	5 (5–5)	4 (4–4)	3 (0–4)
Presentation Triceps MRC mean (std)	5 (0)	3 (1.73)	3.2 (1.69)	1.2 (1.5)
Pre-operative Triceps MRC mean (std)	5 (0)	5 (0)	4 (0)	2.3 (1.8)
**BRACHIAL PLEXUS INJURY AT PRESENTATION**				
Axillary Nerve Only	26	0	0	0
C5 only	5	0	0	0
C5/C6	14	2	0	0
C5/C6/C7	0	1	10	11
Other	0	2	4	2
Mean (std)time injury to surgery (months)	7.3 ± 4.4	5.4 ± 2.4	5.4 ± 1.6	5.5 ± 2.16

### Shoulder Abduction Medical Research Council

Pre-operative shoulder abduction MRC demonstrated no significant difference between all groups (1 to 4). Post-operative shoulder abduction MRC measurements are shown in Table 4 and a comparison across all the four groups demonstrated a statistically significant difference (*p* < 0.05). The authors appreciate MRC grading is a clinical measure reported in whole numbers but for purposes of analyses the above results are reported as both median values with the interquartile range (IR) and mean with standard deviation (STD) (Table 4). All the groups achieved a post-operative median shoulder abduction of ≥ M4. Analyses of mean MRC scores demonstrated both groups 1 and 2 achieved M > 4 with mean values of 4.48 ± 0.67 and 4.25 ± 0.5, respectively. While group 3 came close with a mean of 3.7 ± 1.2, group 4 fell short with a mean of 3.2 ± 1.6, both groups showing a statistically significant reduction in MRC in comparison with group 1 (*p* < 0.05) (Table 4). All other group to group pairwise comparisons did not reach significance.

**Table 4:** Pre- and post-operative shoulder abduction assessments.

	**Group 1**	**Group 2**	**Group 3**	**Group 4**
**PRE-OPERATIVE AND POST-OPERATIVE SHOULDER ASSESSMENTS—MEDIAN (IR)**				
Pre-operative Shoulder Abduction MRC	0 (0–4)	2.5 (1–4)	0 (0–0)	0 (0–4)
Post-operative Shoulder Abduction MRC*	5 (4–5)	4 (4–4.5)	4 (4–4)	4 (3–4)
Pre-operative Shoulder Abduction AROM(°)	50° (20–90)	60° (15–75)	–	–
Post-operative Shoulder Abduction AROM(°)*	170° (85–180)	117.5° (97.5–140)	90° (35–150)	60° (40–155)
**PRE-OPERATIVE AND POST-OPERATIVE SHOULDER ASSESSMENTS—MEAN (STD)**				
Pre-operative Shoulder Abduction MRC	1.44 (1.94)	2.5 (2.1)	0.57 (1.5)	1.1 (1.9)
Post-operative Shoulder Abduction MRC*	4.48 (0.67)	4.25 (0.5)	3.7 (1.2)	3.2 (1.6)
Pre-operative Shoulder Abduction AROM(°)	68.5° (55.5)	50° (31.2)	–	–
Post-operative Shoulder Abduction AROM(°)*	132.7° (54.1)	118.8° (32.8)	93.9° (63.2)	92.5° (62.5)

*(-) insufficient data for analysis*.

(*) Differences between groups 1 vs. 3 and 1 vs. 4 statistically significant (p < 0.05).

The time to post-operative shoulder abduction MRC plateau (Table 5) was not significantly different between groups.

**Table 5:** Post-operative shoulder abduction assessment plateau.

	**Group 1**		**Group 2**		**Group 3**		**Group 4**	
	**Median (IR)**	**Mean (Std)**	**Median (IR)**	**Mean (Std)**	**Median (IR)**	**Mean (Std)**	**Median (IR)**	**Mean (Std)**
Time to plateau MRC (months)	15 (12–29)	22.2 (16.2)	19.5 (16–25)	20.2 (6.1)	18 (10–35)	23.5 (17.5)	19.5 (13–27)	23.5 (14.7)
Time to plateau AROM (months)^*^	14 (9–25)	18.5 (15.2)	18 (11–25)	18 (8.8)	29 (19–55)	33.6 (19.8)	23.5 (19–34)	26.4 (13.8)

* *Differences between groups 1 vs. 3 and 1 vs. 4 statistically significant (p < 0.05)*.

### Shoulder Abduction Active Range of Motion

Pre-operative shoulder abduction AROM demonstrated no significant difference between groups 1 to 4. Mean and median post-operative shoulder abduction AROM measurements are shown in Table 4, and comparison across all the four groups has demonstrated a statistically significant difference (*p* < 0.05). Similar to MRC measurements, key findings on subgroup analyses demonstrated statistically significant differences when comparing group 1 with groups 3 and 4 (*p* < 0.05), while all the other group to group pairwise comparisons did not reach significance.

The time to post-operative shoulder abduction AROM plateau was found to be significantly different when group 1 was compared with groups 3 and 4 (*p* < 0.05) (Table 5).

### Subgroup Analysis

An additional analysis was performed to compare the results of patients who had normal triceps function in the immediate pre-operative setting, irrespective of their presentation triceps strength (groups 1 and 2 patients combined), to patients who had abnormal pre-operative triceps (group 3) who underwent triceps to axillary nerve transfer. These results determined that the patients with normal pre-operative triceps function (combined groups 1 and 2) achieved improved post-operative shoulder abduction MRC and AROM scores compared with those in group 3 with abnormal triceps (*p* < 0.05). In addition to these variables, the combined groups 1 and 2 patients demonstrated a significantly shorter time to AROM plateau (see results in Supplementary Table 1).

The authors recognise the contributions of supraspinatus to shoulder abduction are significant; therefore, a further analysis was conducted to ascertain shoulder abduction outcomes in patients with an intact suprascapular nerve compared with those who also underwent a SAN to SSN transfer in addition to triceps to axillary nerve transfers. Group 1 was the only group with sufficient numbers to allow for this dichotomised statistical analysis (see Supplementary Table 2). The statistical analysis determined that patients in group 1 with an intact suprascapular nerve presented with a significantly improved pre-operative shoulder abduction MRC score compared with those who required a SAN to SSN nerve transfer. However, no other variables demonstrated any significant differences between the groups.

## Discussion

Loss of deltoid function in isolated axillary nerve or brachial plexus injuries results in severe functional deficits for patients, with impairment in their personal and vocational activities. Surgical options for treatment include nerve grafting, nerve transfers, tendon transfers, or shoulder arthrodesis (3). Several groups have reported in small series of patients the successful restoration of deltoid strength using triceps nerve to axillary nerve transfers. While the outcomes are variably reported, most patients achieve ≥ M3 shoulder abduction (3–7, 10, 11). For the purposes of analysis in this study, the authors considered ≥M4 a successful and meaningful functional result.

The group 1 patients who presented with normal functioning triceps demonstrated the best post-operative results after triceps to axillary nerve transfers with a median shoulder abduction M5 and AROM of 170°. The 45 patients in group 1 alone demonstrate the largest reported series of patients receiving triceps to axillary nerve transfers for deltoid reanimation and represent consistently better outcomes compared with the reported literature of the previous case series (1).

The patients in group 2 demonstrate a trend in reduced post-operative shoulder abduction MRC and AROM measurements in comparison to group 1. However, these differences did not reach statistical significance, indicating that in this series good post-operative MRC power may be achieved using an abnormal triceps nerve that clinically recovers completely preoperatively for deltoid reanimation. The use of an abnormal triceps nerve after incomplete clinical recovery (group 3) resulted in a significant reduction in post-operative shoulder abduction MRC and AROM, and an increase in time to AROM plateau in comparison with group 1. The authors hypothesise that there is a reduced axonal count in groups 2 and 3 triceps nerve donors in comparison to the group 1 patients, which is represented by weakness in triceps function at presentation. While group 2 triceps function recovers, group 3 improves but continues to demonstrate abnormal triceps strength, which may account for slightly poorer reinnervation of the deltoid muscle target.

The patients in group 4, with the unsuitable nerve to triceps donors, showed significantly lower post-operative shoulder abduction MRC and AROM scores, and an increase in time to AROM plateau in comparison with group 1. These patient groups in whom nerve to triceps was found to be abnormal or unusable (groups 3 and 4) were found to have a greater extent of injury to their brachial plexus with at least C5/6/7 involvement compared with C5/6 or isolated axillary nerve injuries (groups 1 and 2). The authors propose that due to additionally paralysed shoulder muscles (such as serratus anterior, supraspinatus, infraspinatus, subscapularis, pectoralis, and teres major muscles), the more severely injured groups 3 and 4 patients will predictably have reduced shoulder functional outcomes, a finding that was supported by Rezzadeh et al. in a systematic review of SAN to SSN outcomes (15). This series further illustrates that triceps deficiency can act as a surrogate marker of a more extensive plexus injury and predict poorer outcomes if the weakness persists.

The authors have presented the largest series of triceps nerve to axillary nerve transfers for deltoid reanimation demonstrating successful results across all four sub-groups of patients. However, there are some limitations to this study. Despite the relatively large case series numbers in this field of surgery, the smaller number of patients in groups 2, 3, and 4 along with occasional missing data variables limited some of the statistical analyses (Figure 2). To present data objectively, both mean (with standard error of the mean) and median (with interquartile ranges) were reported, acknowledging each has limitations when applied to smaller groups in this series. While the follow-up rate of 33 months on average reflects a good duration of post-operative monitoring, the follow-up intervals varied and were often very long, reflecting the unfortunate reality of the limited resources within the healthcare system. As a result, it is likely that the times to recovery plateau are overstated. Any analysis of patients with brachial plexus injury is difficult as they are a heterogenous group, and each injury is unique when assessed in detail. Although manual muscle strength testing using the MRC is a relatively well-accepted method of post-operative patient assessment for muscle recovery, the grading system remains a subjective surrogate of an objective measure. The risk of inter-investigator variability was reduced by the senior author conducting an assessment of the MRC grade.

The critical question the authors sought to answer is whether deltoid reanimation should be undertaken using functional but persistently abnormal nerves to triceps, and if so, under what circumstances are the triceps donor nerves still the best option for this group? These questions are addressed below with support from the results of this case series. The decision to proceed with the triceps to axillary nerve transfer in this series was based on a pre-operative triceps strength of ≥M4, as determined by the senior surgeon. This is a key determinant and a variable that is not often recorded or presented clearly in the literature. The senior author then conducted a thorough intraoperative assessment of all separate triceps nerves, which were required to display adequate strength in stimulation as well as redundancy before the decision to transfer was made.

The authors considered deltoid re-animation of ≥M4 as successful for the purpose of this study. Analyses using the median values (Table 4) demonstrate that group 1 achieves this successfully with M5, while median values for groups 2–4 result in M4 power (albeit with decreasing interquartile ranges). The same parameter analysed with mean values demonstrated that groups 1 and 2 achieved ≥M4, while groups 3 and 4 fell short, with the latter groups showing a statistically significant reduction in MRC in comparison with group 1 (*p* < 0.05). Subgroup analysis of the patients in Group 1 who also underwent SAN to SSN transfer compared with those who underwent triceps to axillary nerve transfer alone did not demonstrate differences in post-operative shoulder abduction scores, demonstrating the efficacy of the results of nerve transfer in restoring all damaged components of shoulder abduction.

On further subgroup examination, there were no differences in post-operative outcomes between groups 2 and 3 patients. The trends of improved post-operative MRC and AROM and a shorter AROM time to plateau in group 2 vs. 3 may be explained by improved axonal counts in group 2 where triceps nerve donors recover to normal prior to transfer, rather than persisting as abnormal. These findings are further supported by the subgroup analysis of patients with normal triceps in the pre-operative setting (groups 1 and 2 combined) vs. those with abnormal but usable triceps nerves (group 3), demonstrating improved post-operative shoulder abduction outcomes in the normal triceps groups, likely representing a less severe brachial plexus injury and improved axonal counts in the triceps nerves transferred for deltoid reanimation.

Comparison of groups 3 and 4 patients did not demonstrate any significant differences in post-operative outcomes (Tables 4, 5). When faced with the decision to use an abnormal triceps nerve or an alternative transfer for deltoid reanimation, the surgical priorities for the patient must be considered. These patients commonly fall in the category of C5/6/7 or more severe injuries, thereby extraplexal donors are often required to restore shoulder abduction in addition to other pre-operative deficits. The results in group 4 patients using non-triceps donors for reconstruction were not shown to be superior in achieving deltoid reanimation when compared with using an abnormal triceps nerve donor. The authors thereby conclude that utilising an abnormal triceps nerve that demonstrates sufficient strength and redundancy intraoperatively is preferable to alternative transfers for deltoid reanimation, potentially preserving other extraplexal donors or grafting techniques for other reconstructive needs.

## Conclusion

In conclusion, the current preference of the senior author is to utilise multiple triceps to axillary nerve transfers for restoration of deltoid function even if triceps nerves are observed to have less than normal function, as long as all criteria listed below are met:

1. The triceps power by the time of surgery is clinically ≥M4.
2. Surgical exploration confirms sufficient triceps donor nerve power.
3. Surgical exploration reveals sufficient functioning triceps nerves for both powering the nerve transfer and maintaining adequate triceps strength.

This approach is undertaken with the knowledge that while results for patients in groups 2 and 3 are inferior in post-operative outcomes in comparison to (the ideal) group 1 patients, the observed outcomes are sufficiently successful that these transfers are equivalent or superior in most cases to alternative options used in group 4 patients.

## Data Availability Statement

The raw data supporting the conclusions of this article will be made available by the authors, without undue reservation.

## Ethics Statement

The studies involving human participants were reviewed and approved by St Vincent's Private Hospital East Melbourne. Written informed consent for participation was not required for this study in accordance with the national legislation and the institutional requirements.

## Author Contributions

SF senior author and surgeon for all operative cases presented in this case series. LS and AW data collection, manuscript, and figure preparation. All authors contributed to the article and approved the submitted version.

## Conflict of Interest

The authors declare that the research was conducted in the absence of any commercial or financial relationships that could be construed as a potential conflict of interest.
