# Supplementary material for: Defining the Reliability of Deltoid Reanimation by Nerve Transfer When Using Abnormal but Variably Recovered Triceps Donor Nerves
Source: Front Surg. 2021 Jun 28;8:691545. doi: 10.3389/fsurg.2021.691545 (PMC8273274; doi:10.3389/fsurg.2021.691545)
Supplement: Supplementary file 1 [file Table_1.DOCX]

| **Preoperative and Postoperative shoulder assessments - median (IR)** | | |
| --- | --- | --- |
|  | **Group 1+2** | **Group 3** |
| **Pre-operative Shoulder Abduction MRC** | 1  (0-4) | 0  (0-0) |
| **Post-operative Shoulder Abduction MRC *** | 5  (4-5) | 4  (4-4) |
| **Pre-operative Shoulder Abduction AROM (**°**)** | 50°  (20-90) | - |
| **Post-operative Shoulder Abduction AROM (**°**) *** | 160°  (85-180) | 90°  (35-150) |
| **Time to plateau MRC (months)** | 16  (12-28) | 18  (10-35) |
| **Time to plateau AROM (months) *** | 14  (9-25) | 29  (19-55) |
| **Preoperative and Postoperative shoulder assessments – mean (std)** | | |
|  | **Group 1+2** | **Group 3** |
| **Pre-operative Shoulder Abduction MRC** | 1.64  (1.91) | 0.57  (1.5) |
| **Post-operative Shoulder Abduction MRC *** | 4.46  (0.65) | 3.7  (1.2) |
| **Pre-operative Shoulder Abduction AROM (**°**)** | 66.3°  (53.1) | - |
| **Post-operative Shoulder Abduction AROM (**°**) *** | 132°  (52.5) | 93.9°  (63.2) |
| **Time to plateau MRC (months)** | 22  (15.4) | 23.5  (17.5) |
| **Time to plateau AROM (months) *** | 18.5  (14.7) | 33.6  (19.8) |

**Supplementary Table 1: Pre and Postoperative shoulder abduction assessments combined Group 1 and 2 vs. Group 3**

*(-) insufficient data for analysis*

*(*) Differences between groups 1+2 vs. 3 (p<0.05)*
