# Supplementary material for: Defining the Reliability of Deltoid Reanimation by Nerve Transfer When Using Abnormal but Variably Recovered Triceps Donor Nerves
Source: Front Surg. 2021 Jun 28;8:691545. doi: 10.3389/fsurg.2021.691545 (PMC8273274; doi:10.3389/fsurg.2021.691545)
Supplement: Supplementary file 2 [file Table_2.DOCX]

| **Preoperative and Postoperative shoulder assessments - median (IR)** | | |
| --- | --- | --- |
|  | **Group 1**  **SAN to SSN** | **Group 1**  **No SAN to SSN** |
| **Pre-operative Shoulder Abduction MRC *** | 0  (0-0) | 4  (4-4) |
| **Post-operative Shoulder Abduction MRC** | 4  (4-5) | 5  (4-5) |
| **Pre-operative Shoulder Abduction AROM (**°**)** | 40°  (35-45) | 50°  (20-90) |
| **Post-operative Shoulder Abduction AROM (**°**)** | 160°  (70-180) | 170°  (87.5-180) |
| **Time to plateau MRC (months)** | 20  (10-29) | 14  (12-29) |
| **Time to plateau AROM (months)** | 21  (14-26) | 13.5  (6-18.5) |
| **Preoperative and Postoperative shoulder assessments – mean (std)** | | |
|  | **Group 1**  **SAN to SSN** | **Group 1**  **No SAN to SSN** |
| **Pre-operative Shoulder Abduction MRC *** | 0.17  (0.41) | 4  (0) |
| **Post-operative Shoulder Abduction MRC** | 4.18  (0.87) | 4.64  (0.49) |
| **Pre-operative Shoulder Abduction AROM (**°**)** | 40°  (7.1) | 71.2°  (57.4) |
| **Post-operative Shoulder Abduction AROM (**°**)** | 127°  (58.6) | 135°  (52.9) |
| **Time to plateau MRC (months)** | 20.6  (11.7) | 23.1  (18.4) |
| **Time to plateau AROM (months)** | 22.2  (13.3) | 17  (15.8) |

**Supplementary Table 2: Pre and Postoperative shoulder abduction assessments of Group 1patients who underwent SAN to SSN transfer in addition to triceps to axillary nerve ) (n=13) vs triceps to axillary nerve transfer alone (n=32)**

*(-) insufficient data for analysis*

*(*) Differences between groups 1+2 vs. 3 (p<0.05)*
